# Methods for latent image simulations in photolithography with a polychromatic light attenuation equation for fabricating VIAs in 2.5D and 3D advanced packaging architectures

**DOI:** 10.1038/s41378-021-00266-x

**Published:** 2021-05-25

**Authors:** Daniel C. Smallwood, Paul McCloskey, Cian O’Mathuna, Declan P. Casey, James F. Rohan

**Affiliations:** 1 MicroNano Systems Centre, Tyndall National Institute, University College Cork, Lee Maltings, Cork, T12 R5CP Ireland; 2 School of Engineering, University College Cork, College Road, Cork, Ireland

**Keywords:** Nanowires, Electronic devices, Computational nanotechnology, Applied optics, Electrical and electronic engineering

## Abstract

As demand accelerates for multifunctional devices with a small footprint and minimal power consumption, 2.5D and 3D advanced packaging architectures have emerged as an essential solution that use through-substrate vias (TSVs) as vertical interconnects. Vertical stacking enables chip packages with increased functionality, enhanced design versatility, minimal power loss, reduced footprint and high bandwidth. Unlocking the potential of photolithography for vertical interconnect access (VIA) fabrication requires fast and accurate predictive modeling of diffraction effects and resist film photochemistry. This procedure is especially challenging for broad-spectrum exposure systems that use, for example, Hg bulbs with g-, h-, and i-line UV radiation. In this paper, we present new methods and equations for VIA latent image determination in photolithography that are suitable for broad-spectrum exposure and negate the need for complex and time-consuming in situ metrology. Our technique is accurate, converges quickly on the average modern PC and could be readily integrated into photolithography simulation software. We derive a polychromatic light attenuation equation from the Beer-Lambert law, which can be used in a critical exposure dose model to determine the photochemical reaction state. We integrate this equation with an exact scalar diffraction formula to produce a succinct equation comprising a complete coupling between light propagation phenomena and photochemical behavior. We then perform a comparative study between 2D/3D photoresist latent image simulation geometries and directly corresponding experimental data, which demonstrates a highly positive correlation. We anticipate that this technique will be a valuable asset to photolithography, micro- and nano-optical systems and advanced packaging/system integration with applications in technology domains ranging from space to automotive to the Internet of Things (IoT).

## Introduction

Photolithography is a process whereby a photosensitive film, or photoresist, is exposed to light. Light propagation prediction and modeling enable ambitious photomask designs for film patterning that drive the cutting edge of 2.5D and 3D advanced packaging architectures with increased functionality, enhanced design versatility, reduced power consumption, small form factor and high bandwidth^1–7^. These qualities are essential for next-generation technologies in domains such as high-end computing, mobile devices, radio frequency (RF), automotive, space, artificial intelligence (AI), biotechnology and the Internet of Things (IoT)^4,8–11^.

In 2.5D architectures, an array of chips is bonded to an interposer^12^. An interposer is an insulating substrate for I/O redistribution comprising vertical interconnect access (VIA) conductive materials, such as Cu, that interconnect the top and bottom surfaces to form through-substrate vias (TSVs)^13^. In 3D architectures, successive chips are bonded to one another in a vertical stack with orientations such as front-to-front, front-to-back and back-to-back, where each chip comprises VIAs that connect the top and bottom surfaces^12,14^.

**Fig. 1:**
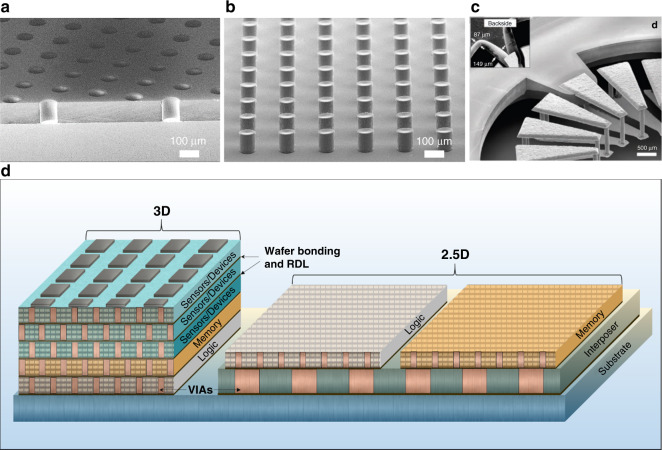
VIAs in 2.5D and 3D advanced packaging architectures. **a** Relief mold array in THB-151N photoresist for bottom-up electroplating of Cu VIAs. **b** Electroplated Cu VIA array from **a**. **c** Angled view of a toroidal microinductor device using VIA technology to create stacked inductor windings (Reprinted without modification from ref. ^16^ under the Creative Commons Attribution 4.0 International License). **d** 2.5D and 3D advanced packaging architectures. The x-z cross-section reveals an array of Cu VIAs that interconnect the top and bottom surfaces of both the chips and the interposer. The chips are integrated in a single stack in 3D, whereas they are placed side by side in 2.5D. A redistribution layer (RDL) is depicted between successively stacked chips and between the chips and the substrate/interposer. The depicted VIAs are similar in size to the example Cu VIAs in (**b**) (the same order of magnitude). The microinductor device in (**c**) could be implemented as a discrete layer in the 3D stack to form a power supply in package (PwrSiP) or together with the sensors to form a power supply on chip (PwrSoC)^52^

Figure 1a shows a photoresist relief mold array for the bottom-up electroplating of Cu VIAs. The corresponding electroplated Cu VIA array is shown in Fig. 1b. This VIA array can be used in a build-up interposer in 2.5D, wherein the interposer substrate comprises an elastic material with a low Young’s modulus, such as an acrylic polymer. This elastic material reduces or eliminates material stress induced by a mismatch in the coefficient of thermal expansion (CTE) between adjacently packaged devices. Furthermore, this VIA array could be used for 3D packaging in via-middle and via-last complementary metal oxide semiconductor (CMOS) processing windows to create, for example, high bandwidth memory (HBM)^1,12^. In addition, VIAs can be monolithically integrated with sensors and devices to form internal or external device/sensor components. For example, in Fig. 1c, an air-core microinductor device fabricated in silicon uses internal VIAs to enable stacked conductor layers in a monolithic format, which can be readily integrated into 2.5D and 3D architectures^15,16^. Microinductor VIAs dramatically reduce the passive device form factor to enable on-chip, granular point-of-load (PoL) power delivery, which will be an essential feature of future microprocessors, complex systems on chip (SoCs) and emerging fully autonomous microelectromechanical systems (MEMS) devices^17,18^. Figure 1d depicts a cross-section of 2.5D and 3D advanced packaging architectures to demonstrate the versatility of Cu VIA arrays.

**Fig. 2:**
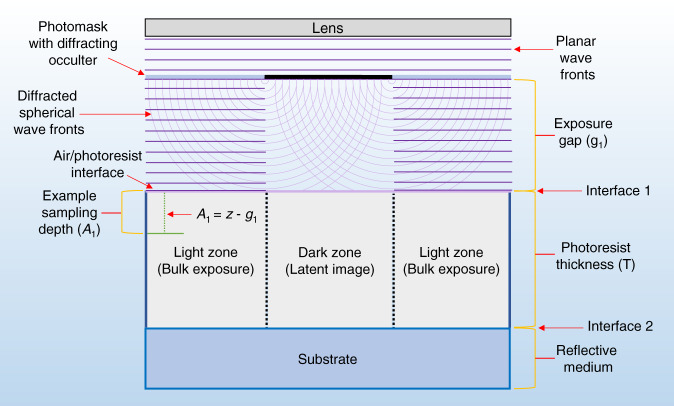
Schematic depicting Huygens-Fresnel diffraction of a planar wave front at a photomask occulter boundary. Planar waves diffract into spherical waves at the occulter boundary, where the diffracted wavefronts are superimposed in the figure as concentric rings (x-z cross-sections of concentric spheres). Spherical waves initiate photochemical reactions in the dark zone, distorting the target latent image. Diffraction is a function of boundary geometry, boundary spacing and the incident wavelength to boundary size ratio. Latent image distortion is a function of diffraction, exposure gap length, film thickness, film photosensitivity and various photoresist processing parameters, including baking and etching. We note that this schematic can also represent general optical systems comprising stacks of attenuating and non-attenuating layers. The parameters *g*_1_, *A*_1_, and *T* are referred to throughout this paper

In photolithography, light exposure initiates chemical reactions at selective sites in a photoresist film, creating a latent image pattern, or an invisible array of shapes that is subsequently rendered visible by photoresist development^19^. An example exposure process for VIA mold formation is depicted in Fig. 2.

The photochemical reaction initiates from the top downwards (or from the bottom upwards in backside exposure), resulting in a depth-dependent exposure dose along the thickness of the photosensitive film. For latent image formation, the exposure dose at the photoresist-substrate interface must be sufficient for the photochemical reaction to fully proceed. Ideally, the photochemical reaction only occurs in the light zone and is completely blocked in the dark zone. However, in practice, the design pattern is often degraded by diffraction at the aperture and occulter boundaries, causing spherical wavefronts to constructively interfere and initiate undesired photochemical reactions in the target dark zone.

Photoresist latent image simulation thus requires three main quantitative components: (1) the depth-dependent exposure dose along the thickness of the photosensitive film, (2) the critical exposure dose, which is the energy threshold past which the photochemical reaction has fully proceeded, and (3) the intensity profile underneath a photomask diffractor.

In this paper, we focus on light propagation in photolithography for VIA mold latent image simulations. These simulations require parameters for diffraction, reflection, transmission and attenuation. Furthermore, accurate modeling demands a coupling between light propagation phenomena and photochemical behavior. Most reported latent image simulation methods use the paraxial approximation for diffraction equations. This approximation introduces significant error for off-axis points in the near aperture/occulter condition, the exception being a long rectangular diffractor. Consequently, many latent image simulation papers report formulae for aperture geometries, including an infinite straight edge^20^, a single long slit^21–24^ and a rectangle^25–28^, while robust simulation platforms use a range of formulae, including versions of the Fresnel, Kirchoff and Rayleigh-Sommerfeld diffraction integrals^29–33^. Reported methods require rectangular geometries in the ultra-near aperture/occulter condition, where the Fresnel number, F, is much greater than one, as is commonly observed in photolithography. A method for a circular geometry at this distance will be highly useful for VIA mold latent image simulation, as the conventional VIA shape is circular^6,12,13^.

The Dill method^34^ and the enhanced Dill method^35^ are frequently used to determine exposure dose information. These methods and several variants thereof are applicable to monochromatic exposure systems^23–28,36–39^, while some other variants use a lumped analysis technique for polychromatic exposure^21,22,40^ that could be suitable, for example, in broad-spectrum Hg bulb exposure conditions (g-, h- and i-line). These methods enable the determination of exposure dose information, but they are only suitable when a photoresist bleaches upon exposure, meaning a proportionality between resist transparency and exposure duration is required. In addition, these methods require rigorous metrology and characterization^41^, which incurs cost and takes time to perform.

We identify a need for a method to enable VIA mold latent image simulations for both monochromatic and polychromatic exposure systems that can be performed prior to photoresist processing. This method must be fast, accurate and applicable for any photoresist. This will add great value by: (1) enabling accurate prediction of photolithographic patterning in 2D and 3D geometries for either monochromatic or polychromatic exposure conditions, (2) enabling testing of any number of photoresists before purchase and delivery, (3) avoiding unnecessary wait times for vendor supply chains, (4) eliminating unnecessary cost and time consuming laboratory work, (5) bypassing long machinery use queues in busy processing facilities, and (6) enabling 24/7 remote access to safely trial unlimited experiments from any location, which is becoming an ever more important need in the modern world.

Herein, we provide a fast and accurate method for VIA mold latent image simulation that is available prior to photoresist processing, is valid for both monochromatic and polychromatic exposure systems and is applicable for any photoresist. We propose a succinct calculation method that features a complete coupling between light propagation phenomena and photochemical behavior. We present this method in the form of a single equation for convenience and ease of use. In addition, we use a fast and exact scalar diffraction equation that produces accurate solutions for both on- and off-axis observation points for circular aperture/occulter geometries, which is valid in the ultra-near field. While we focus on photolithography for VIA mold fabrication, we present our polychromatic light attenuation equation and our photoresist exposure methods in a general form for broad application in photolithography and optical systems.

We perform our simulations and experiments with THB-151N, a negative tone photoresist comprising acrylate monomers and photosensitizers dissolved in a PGMEA solvent. This i-line sensitive resist fully cross-links upon exposure, is highly viscous and well suited for rapid thick film processing (e.g., 50+ µm), as it does not require a post-exposure bake and is resistant to acidic electroplating baths. An example polymerization mechanism involves free radicals created during UV exposure reacting with dissolved acrylate monomers to form a highly cross-linked polymeric matrix^42^.

## Results

### Polychromatic light attenuation in a simple new equation

We first derive a simple formula for polychromatic light attenuation. We present this versatile new equation in a generalized form that is valid for single material layers through to stacks of attenuating and non-attenuating media. To do this, we begin with the Beer-Lambert law for light attenuation, as provided by Eq. (1), where *α* is the absorption coefficient, *I*_0_ is the bulb irradiance, z is the attenuating path length and *I* (z) is the luminous intensity^36–38,43^.

1 $$I\left( z \right) = I_0e^{ - \alpha z}$$

Equation (1) neglects reflection and is only sufficient for systems with a single attenuating layer. An improved formulation is Eq. (2), which is valid for multiple layers and includes reflection. Monochromatic wavelength species *λ*_*i*_ is z distance from the source, and the gap-modified z-factors, *A*_1_ (Eq. (3)) and *A*_2_ (Eq. (4)), compensate for a non-light attenuating medium somewhere along z. As shown in Fig. 2, *A*_1_ is the transmitted attenuating path length, and *A*_2_ is the internally reflected attenuating path length, where *T* is the film thickness and *g*_1_ is the non-attenuating path length. *R*_*i*1_ and *R*_*i*2_ are the reflection coefficients at interfaces 1 and 2, respectively, as shown in Fig. 2, which are calculated with Fresnel’s equations.

2 $$I_i\left( z \right) = (1 - R_{i1})I_0\left[ {e^{ - \alpha _iA_1} + R_{i2}e^{ - \alpha _iA_2}} \right]$$

3 $$A_1(z) = z - g_1$$

4 $$A_2\left( z \right) = 2T - A_1 = 2T - z + g_1$$

To enable calculations for polychromatic light attenuation, a novel method using the aerial (or unattenuated) relative intensity spectrum of the light source is now introduced. To aid comprehension, we have included Eq. (5), which calculates the attenuated light intensity fraction of a monochromatic wavelength species *λ*_*i*_ at a distance z from a polychromatic source. Internal reflection, *R*_*i*2_, has been omitted in this example formula for clarity but is later included in our final formulation. The new parameter, *I*_*λi*_, is the aerial relative intensity fraction of the species *λ*_*i*_. This parameter is determined from the intensity spectrum of the source, as provided by the manufacturer, or as measured in situ with a photometer.

5 $$I_{i_{A_1}}(z) = (1 - R_{i1})I_0I_{\lambda _i}e^{ - \alpha _iA_1}\frac{{e^{ - \alpha _iA_1}}}{{\mathop {\sum }\nolimits_{i = 1}^n I_{\lambda _i}e^{ - \alpha _iA_1}}}$$

As an example, the broad-spectrum USH-250D super high pressure UV-type mercury lamp used in our experiments has g-, h-, and i-line relative intensities of 0.76, 0.49, and 1.0, respectively. Therefore, we can be certain that if we measure the unobstructed total aerial (unattenuated) broad-spectrum intensity with a new bulb, that it comprises 33.8% g-line, 21.8% h-line, and 44.4% i-line, which occurs in the attenuation factor in (5) at *z* = *g*_1_ (interface 1), as required. Inside the attenuating medium, the wavelengths attenuate according to their absorption coefficients, which is accounted for by the formula.

Comprising the sum of all $I_{i_{A_1}}$, Eq. (6) calculates the transmitted intensity from a polychromatic source at any depth in an attenuating medium by using the first gap-modified z-factor, *A*_1_. Equation (7) calculates the internally reflected intensity with *R*_*i*2_ and the second gap-modified z-factor, *A*_2_. The total polychromatic attenuated intensity is provided by Eq. (8). This formula is applicable to polychromatic systems involving single materials through to layered stacks comprising highly variable material properties.

6 $$I_{A_1}(z) = I_0\frac{{\mathop {\sum }\nolimits_{i = 1}^n I_{\lambda _i}e^{ - 2\alpha _iA_1}(1 - R_{i1})}}{{\mathop {\sum }\nolimits_{i = 1}^n I_{\lambda _i}e^{ - \alpha _iA_1}}}$$

7 $$I_{A_2}(z) = I_0\frac{{\mathop {\sum }\nolimits_{i = 1}^n I_{\lambda _i}e^{ - 2\alpha _iA_2}(1 - R_{i1})R_{i2}}}{{\mathop {\sum }\nolimits_{i = 1}^n I_{\lambda _i}e^{ - \alpha _iA_2}}}$$

8 $$I_t(z) = I_{A_1} + I_{A_2}$$

The Beer-Lambert law (Eq. (1)) has previously been used in photolithography to calculate monochromatic light attenuation using a standard formulation^44,45^ and a non-standard version that accounts for a change in the photoresist absorption coefficient during exposure^37,38^. As these pre-existing formulae only account for a single wavelength, they are incompatible with polychromatic light. Our Eq. (8) accounts for multiple wavelengths and is thus compatible with polychromatic exposure systems. This is made possible by including for the first time, terms comprising: (1) the relative intensity of each of the incident wavelengths in polychromatic exposure systems and (2) the corresponding absorption coefficient of each wavelength. This equation enables easy calculation and modeling of polychromatic light attenuation for micro- and nano-optical systems.

### Advancing methods in photolithography: depth-selective exposure dose and critical exposure dose determination

We now demonstrate the effectiveness of our polychromatic light attenuation equation with novel methods in photolithography. The standard exposure energy metric in photolithography is the aerial (unattenuated) exposure dose, typically in mJ/cm^2^, as provided by Eq. (9), where *t* is the exposure time in seconds and *I*_0_ is the bulb irradiance in mW/cm^2^. Inserting this into Eq. (2) yields Eq. (10), which describes the attenuated exposure dose of the wavelength species *λ*_*i*_ at a distance z from the photomask.

9 $$ED = tI_0$$

10 $$ED_i(z) = ED(1 - R_{i1})\left[ {e^{ - \alpha _iA_1} + R_{i2}e^{ - \alpha _iA_2}} \right]$$

Equation (10) is only applicable to monochromatic light. This equation poorly accounts for broad spectrum exposure due to its exponential dependence on *α*_*i*_, which can vary by one or more orders of magnitude when moving from g- to i-line (*λ*=436 nm to *λ*=365 nm).

To account for broad spectrum exposure, we insert Eq. (9) into our new attenuation Eq. (8) to produce Eq. (11).

11 $$ED(z) = tI_t$$

This formula enables quick calculation of the exposure dose (ED) at any thickness in a photoresist film. To determine if the ED is sufficient to trigger the desired photochemical reaction, it must be compared to the critical exposure dose (CED) (Eq. (12)). This comparison is essential for all types of photolithography, especially for modulated exposure^30,46^, as it enables determination of cross-linked sites in negative resist and soluble sites in positive resist. *ED*_*spec*_ is the specified aerial ED at a given photoresist thickness, as provided in the photoresist technical data sheet (TDS), or as determined from in situ experiments. $I_{A_{1,b,T}}$ and $I_{A_{2,b,T}}$ are the transmitted and internally reflected (or substrate reflected) intensity contributions using *b*, the bulb relative intensity spectrum, as previously discussed, and *T*, the film thickness.

12 $$CED = ED_{spec}\left( {\frac{{I_{A_{1,b,T}} + I_{A_{2,b,T}}}}{{I_0}}} \right)$$

### Exact and fast scalar diffraction equations for the ultra-near field

We now introduce exact and fast scalar diffraction equations that are approximation-free and derived directly from the Rayleigh-Sommerfeld integral. Equations (13) and (14) are suitable for circular apertures and occulters, respectively, and Eq. (15) is the corresponding geometric parameterization^47^. *U*_*A,C*_ stands for the amplitude of the incident light, *U*, for an aperture, *A*, or occulter, *C*. The coefficient *U*_0_ is the square root of the bulb aerial intensity, *k* is the wavenumber for the first stacked layer, *x* is a radial point on the observation plane, *r* is the radius and z is the distance from the diffractor.

13 $$U_A\left( {x,y,z} \right) = U_0\left[ {e^{ikz} - \frac{z}{{2\pi }}{\int}_0^{2\pi } {\frac{{e^{ik\sqrt {z^2 + c^2(\varphi )} }}}{{\sqrt {z^2 + c^2(\varphi )} }}d\varphi } } \right]$$

14 $$U_C\left( {x,y,z} \right) = - \frac{{U_0z}}{{2\pi }}{\int}_0^{2\pi } {\frac{{e^{ik\sqrt {z^2 + c^2(\varphi )} }}}{{\sqrt {z^2 + c^2(\varphi )} }}d\varphi }$$

15 $$c\left( \varphi \right) = x_i\,cos\varphi + \sqrt {r^2 - x_i^2\,sin^2\varphi } ,\left( {i = 1,2, \ldots ,n} \right)$$

The radial point step size, *x*_*i*_ in Eq. (15), is arbitrarily specified, which sets the resolution of Eqs. (13) and (14), adding great versatility to the equations. Accurate calculation results are obtained even when the step size is greater than the diffracting wavelength(s). This result is due to the integral in Eqs. (13) and (14), which explores the region of interest with the infinitesimal angular increment, *dφ*, as corresponds to infinitesimal arc lengths (e.g., <<405 nm). Moreover, circular symmetry enables full 2D cross-sections of the observation plane, which can be further stacked to create 3D volumetric maps.

Equations (13) and (14) are easily enabled for photolithography by inserting *I*_*t*_ from Eq. (8) to create Eq. (16).

16 $$I_{A,C}\left( {x,y,z} \right) = I_t\left| {U_{A,C}} \right|$$

We note that depending on the photoresist absorption coefficients and thickness, the internally (or substrate) reflected intensity contribution, $I_{A_2}$ from Eq. (8), can sometimes be neglected. When this parameter is neglected, it reduces the computational load of Eq. (16) by more than half and is highly recommended if the dose calculation error margin is sufficiently small. For example, in CMOS photolithography, very thin films are typically used (e.g., 18 nm half-pitch in 2020)^1^. With high-power exposure from the top downwards for high-throughput manufacturing, the incident wavelengths attenuate little along the thickness of the photoresist film such that the intensity reflected from the substrate is significant. However, in photolithography for VIA fabrication, thick films are used (e.g., 50+ µm) that significantly attenuate the incident light along the photoresist thickness. Furthermore, with an optimized exposure procedure, the ED at the photoresist/substrate interface equals the minimum CED threshold. Under these conditions, the reflected intensity is minimal and has little effect on the latent image geometry. For example, the CED of THB-151N is 23.9 mJ/cm^2^ when including $I_{A_{2,b,T}}$ and 23.6 mJ/cm^2^ without $I_{A_{2,b,T}}$ at *T* = 100 µm, which is a difference of only ~1% at the substrate level, which is the point of maximum of divergence.

We provide several additional near-field diffraction formulae in the Supplementary Information. These formulae converge quickly, are applicable for various diffractor geometries, and include additional terms for refraction and angled incidence. We include some useful examples of lithography applications using these additional equations, and we discuss their range of applicability with an analysis of the approximations made in their derivation (e.g., the paraxial approximation).

### Equation validation: simulations and experiments

To verify Eqs. (8), (11), (12), and (16), we first use 2D and 3D latent image simulations to make three predictions. Next, we validate our predictions by comparing latent image simulations to directly corresponding experimental data. Our simulations were performed in Wolfram Mathematica^48^ with Eq. (16) formulated for an occulter and polychromatic exposure. We used THB-151N photoresist material parameters and attenuation factors for the g-, h- and i-lines, which are characteristic of Hg bulbs.

**Fig. 3:**
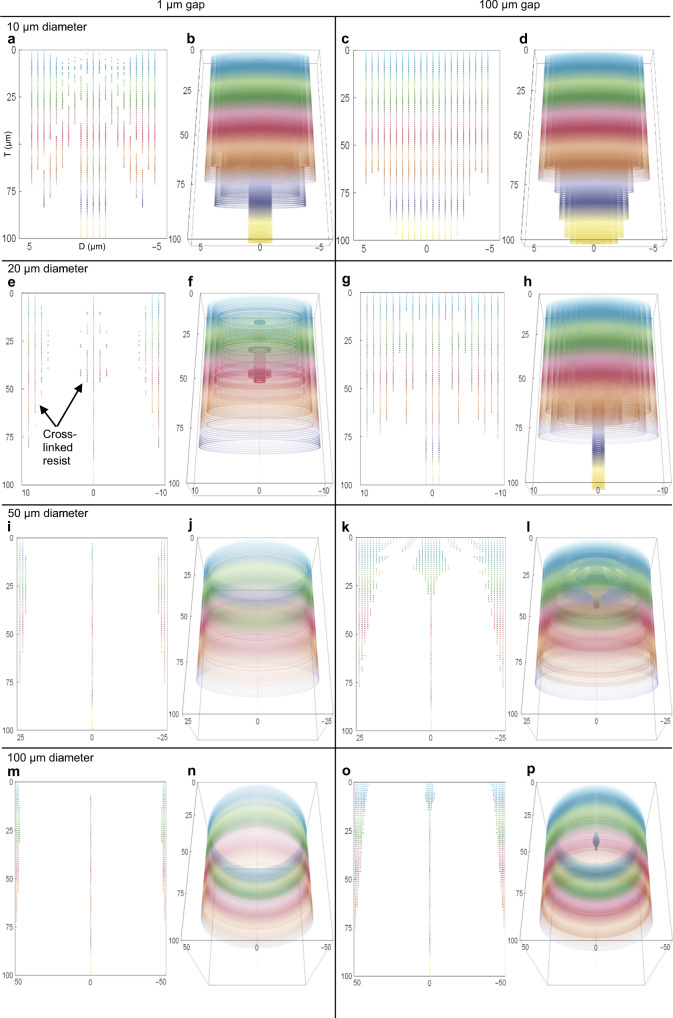
Latent image simulations of varying photomask occulter diameters and air gaps. The ordinate is the top downwards photoresist thickness, T, (µm), and the abscissa is the feature diameter, D, (µm). The unobstructed aerial ED = 1700 mJ/cm^2^, diffraction λ=405 nm and the simulation resolution is 0.5 µm for (**a**–**d**) and 1 µm for (**e**–**p**). The figure color scales with photoresist thickness to differentiate between height levels, where colored data points represent sites of cross-linked photoresist. Rows 1–4 correspond to 10, 20, 50, and 100 µm diameter circular occulters, respectively. Columns 1–2 and 3–4 correspond to air gaps of 1 and 100 µm. **a** 2D x-z photoresist cross-section with D=10 µm and g=1 µm. Light intensity calculation time (t) = 2 s. **b** 3D wrap-around view of (**a**) with a z-axis of rotation at x=0 and y=0. **c** 2D cross-section with D=10 µm, g=100 µm and t = 1 s. **d** 3D wrap-around view of (**c**). **e** 2D cross-section with D=20 µm, g=1 µm and t = 5 s. **f** 3D wrap-around view of (**e**). **g** 2D cross-section with D=20 µm, g=100 µm and t = 1 s. **h** 3D wrap-around view of (**g**). **i** 2D cross-section with D=50 µm, g=1 µm and t = 37 s. **j** 3D wrap-around view of (**i**). **k** 2D cross-section with D=50 µm, g=100 µm and t = 15 s. **l** 3D wrap-around view of (**k**). **m** 2D cross-section with D=100 µm, g=1 µm and t = 1 m 41 s. **n** 3D wrap-around view (**m**). **o** 2D cross-section with D=100 µm, g=100 µm and t = 1 m 1 s. **p** 3D wrap-around view of (**o**)

Figure 3a–d shows a 10 µm diameter mold in 100 µm thick THB-151N, which corresponds to an aspect ratio (AR) of ten. Extensive diffraction effects are present, where full cross-linking occurs with both a 1 µm and a 100 µm air gap. Figure 3e–h shows a 20 µm diameter mold in 100 µm thick THB-151N (AR=5). This mold is significantly affected by diffraction, where full cross-linking occurs with a 100 µm air gap. Figure 3i–l shows that a 50 µm diameter mold is feasible with a 1 µm gap (AR=2), but not with a 100 µm gap. A critical threshold is passed when transitioning to a 100 µm diameter mask feature (AR=1), as shown in Fig. 3m–p, where even with a 100 µm air gap, only minimal edge broadening is observed at the mold entrance.

We now make three predictions: (1) an AR of 1 is feasible in a standard broad-spectrum exposure of thick THB-151N photoresist, (2) development difficulty increases proportionally to mold AR and exposure gap length, and (3) all mold epicenters display a column of cross-linked resist that extends along the entire film thickness, which is due to the well-known Arago spot. We recall that the Arago spot is a luminous region in the epicenter of a circular shadow caused by the constructive interference of diffracted light rays with identical path lengths from a circular boundary.

**Fig. 4:**
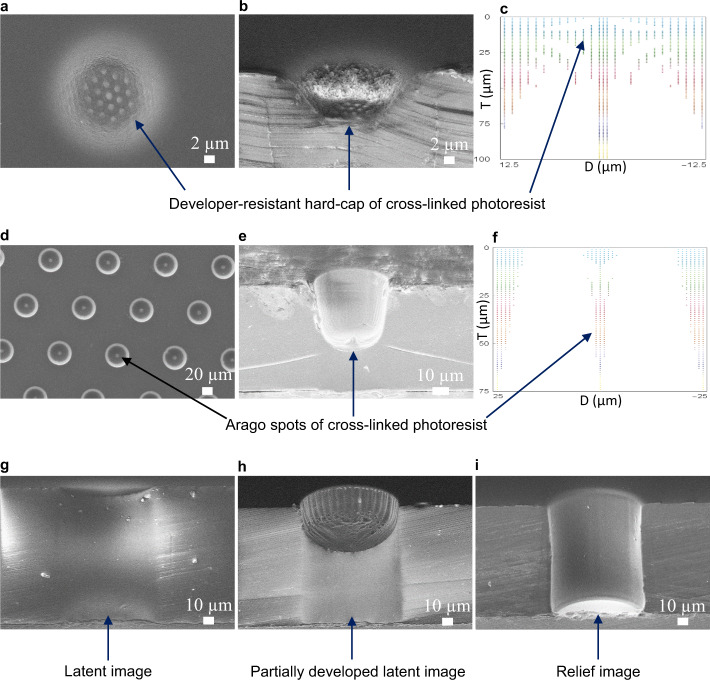
Comparison between experimental and simulation work. **a** Top-down SEM micrograph of a 25 µm target diameter photoresist feature hole displaying a developer-resistant hard cap. **b** 68° angled cross-section of (**a**). **c** Simulated latent image of (**b**), also displaying a hard cap, with a simulation resolution of 1 µm, ED = 1700 mJ/cm^2^, diffraction λ = 405 nm and a 50 µm air gap. The ordinate is the top-down photoresist thickness, T, (µm), and the abscissa is the feature diameter, D, (µm). Light intensity calculation time (t) = 2 s. **d** Top-down view of an array of 50 µm target diameter photoresist feature holes demonstrating Arago spots at the feature epicenters. **e** 68° angled cross-section view of (**d**) with a photoresist thickness of 75 µm. **f** Simulated latent image of (**e**) with D=50 µm, g=50 µm, ED=1100 mJ/cm^2^ and t = 19 s; all other parameters are as previously described. **g** 68° angled cross-section displaying the actual latent image of a 100 µm target diameter feature hole with a photoresist thickness of 125 µm and an exposure gap of 100 µm, as corresponds to simulation Fig. 3k, l. **h** Partially developed latent image of (**g**). **i** Fully developed relief image of (**g**) after wet etching for 90 s in a stock TMAOH developer solution

We now compare our simulation results to directly corresponding experimental data. Figure 4a, b shows scanning electron microscope (SEM) images of a 25 µm target diameter photoresist mold exposed in 100 µm thick THB-151N (AR=4) with a photomask occulter gap length of 50 µm. These images display a “hard cap”, or a highly cross-linked region of photoresist that is extremely resistant to developer solution. This result is predicted by the corresponding latent image simulation (Fig. 4c), wherein a hard cap is clearly visible as a densely packed set of colored data points at the mold entrance.

Figure 4d, e shows partially developed latent images of 50 µm target diameter resist molds exposed with a gap of 50 µm and a film thickness of 75 µm. The epicenters of these molds prominently display the Arago spot, which is also seen in the corresponding latent image simulation (Fig. 4f). This latent image will easily develop under standard conditions with a simple two-part mechanism comprising: (1) the Arago spot creates a thin, ultra-high AR developer-resistant column of resist that nonetheless develops due to structural instability, and (2) throughout mold development, a low AR transient developer-resistant bump is expected, as seen in Fig. 4d, e.

Figure 4g–i shows a 100 µm target diameter photoresist mold exposed with a 100 µm air gap, which transitions from a full latent image to a partially developed relief image and then to a full relief image, respectively. These molds develop with ease (90 s), as predicted by the corresponding latent image simulation in Fig. 3k, l.

Figure 4g–i clearly demonstrates that an AR of 1 is feasible in thick THB-151N, validating prediction 1. When taken together, Fig. 4a–i demonstrates an obvious proportionality between development difficulty and mold AR, validating prediction 2. Finally, in Fig. 4d–f, a developer-resistant bump is depicted at the epicenter of the top-down latent image arrays, cross-sections and simulations, thus validating our final prediction.

We observe in Figs. 3–4 that diffraction effects are highly significant across the entire photomask diffractor, even when the incident wavelengths and diffractor sizes differ by more than two orders of magnitude. For example, significant latent image resolution-degrading effects are present in Fig. 3g, h with an h-line (405 nm) diffracting wavelength and a 50 µm diameter photomask occulter. This striking result is counter to conventional expectation, which assumes that diffraction effects only cause edge broadening and are otherwise negligible at this size ratio. As an example, traditional calculations for photolithographic resolution, herein defined as the smallest printable feature size in a mask aligner, use the well-known formula (Eq. (17))^20,49,50^,

17 $$d = \frac{3}{2}\sqrt {\lambda \left( {g + \frac{T}{2}} \right)}$$

where *d* is the edge-broadening magnitude, *λ* is the incident wavelength, *g* is the air gap and *T* is the photoresist thickness. This equation states that while holding *λ* and *T* constant, the resolution degrading factor, *d*, can be minimized by reducing or eliminating the air gap. As our results show, this statement is correct; however, diffraction effects across the entire target latent image are also highly significant and must be included. Our new succinct latent image formulae offer a significant advancement over this pre-existing equation, as they comprise a complete coupling between light propagation phenomena (including diffraction) and photochemical behavior.

## Discussion

To enable predictive modeling of polychromatic exposure (e.g., with Hg bulbs) of photoresist films for VIA fabrication, we derived a polychromatic light attenuation equation from the Beer-Lambert law. Our equation features novel components comprising: (1) the relative intensity of each of the incident wavelengths in broad-spectrum exposure and (2) the corresponding absorption coefficient of each wavelength. These components enable novel methods and equations for photoresist ED and CED determination that can be used to produce accurate VIA latent image simulations by use of an exact scalar diffraction equation for circular diffractor geometries in the ultra-near aperture/occulter condition (F>>1). While we demonstrate our method in proximity lithography, it could also be adapted for use in a high numerical aperture condition for projection lithography systems. This would require a diffraction formula that is accurate for off-axis points, such as the provided Rayleigh-Sommerfeld scalar diffraction equations (Eqs. (13) and (14)), or any suitable vector diffraction formulae.

Our study demonstrates that accurate predictive modeling of diffraction effects is critically important for VIA fabrication in photolithography. Light diffraction can cause extensive undesired cross-linking underneath photomask occulters, leading to a hard cap that prevents relief mold formation. For broad-spectrum exposure, the hard cap effect could be minimized by using a long-pass filter to selectively eliminate short wavelengths that otherwise quickly absorb at the photoresist surface^44^. In addition, diffraction effects can be reduced by minimizing the exposure gap, as demonstrated in Fig. 3. Alternatively, a shorter exposure wavelength (e.g., with monochromatic exposure) could be used that diffracts less due to a reduced wavelength to diffractor size ratio, which would require a photoresist with a suitable absorption characteristic.

Our equations converge quickly on a standard modern computer. For example, when using parallelization and circular symmetry, Eq. (16) that we used to produce Fig. 3 calculated as much as 388–900x faster than other reported methods for 3D photoresist light intensity simulations^25,51^. The calculation time can be further reduced by increasing the step size between successive radial points, *x*_*i*_, in Eq. (15); however, this step size should remain sufficiently small to enable accurate mapping of the region of interest, wherein a minimum mold diameter to step size ratio (D:SS) can be verified and thereafter used as a standard. The minimum D:SS is 25:1 in Fig. 4, which is shown to be sufficient to produce accurate results by comparing latent image simulations to directly corresponding experimental work, with a highly positive correlation. Therefore, as a general rule, a D:SS of ~25:1 with an SS not exceeding 1 µm is recommended. Furthermore, circular symmetry enables full 2D cross-sections of the observation plane, wherein the grid density increases with inverse proportion to the wrapping angle step size, which can be made as small as desired without affecting the light intensity calculation time. A further note regarding the overall speed of our method is that it negates the need for time-consuming in situ metrology, which can also speed up the simulation process.

These qualities make our technique highly accessible to photolithography practitioners, whether in research or in manufacturing. Potential applications for our technique include (1) numerical modeling with computational software such as Wolfram Mathematica, (2) integration into pre-existing photolithography simulators to broaden their computational domain by adding to their input space, and (3) development of a simple app for on-the-go use in mobile devices. VIA fabrication is expected to become increasingly important as demand grows for 2.5D and 3D advanced packaging architectures. Our equations and methods leverage photolithography to assist in meeting this demand, which could be a valuable asset to emerging advanced packaging technologies.

## Materials and methods

A 100 mm diameter substrate was used with a thickness cross-section from the basal layer upwards of 525 µm Si, 250 nm SiO_2_, 20 nm Ti, and 200 nm Cu. The substrate was first submerged for 30 s in a 150 mm x 25 mm glass petri dish from BRAND (Wertheim, Germany) containing a 10:1 volumetric dilution of DI water:S20 Cu cleaner, which is a solution comprising surfactants and sulfuric acid from Schlötter Ireland DAC (Kildare, Ireland). The substrate was then rinsed in DI water, dried with a N_2_ gun, baked on a hot plate for 5 min at 110 °C to evaporate residual surface-adsorbed H_2_O molecules, and then cooled to room temperature (RT). Next, a Laurell (PA, USA) alignment tool was used to attach the basal layer of the substrate to a 45 mm diameter press-on mount vacuum chuck in a WS-650MZ-23NPPB Laurell photoresist spinner to prepare for two spins. All photoresist processing was carried out under ambient yellow light to prevent stray UV radiation from causing undesired photoresist cross-linking.

Prior to the first spin, RT THB-151N photoresist from JSR Micro NV (Leuven, Belgium) was manually dispensed onto the top Cu layer, and bubbles were removed by suctioning with a disposable pipette to ensure spin uniformity. The first spin comprised six steps: (1) 300 rpm for 25 s to spread out the photoresist, (2) 0 rpm for 30 s to enable reflow, (3) 300 rpm for 10 s as an initial ramp speed, (4) 1100 rpm for 30 s as the thickness-determining step, (5) 300 rpm for 30 s to enable edge bead removal (EBR) with 15 mL of AZ EBR Solvent from Microchem GmbH (Ulm, Germany) dispensed through a pressure-actuated automatic syringe with a 1 mm diameter aperture aimed 7 mm from the substrate edge to ensure suitable bead removal, and finally (6) 1000 rpm for 2 s to level off the photoresist. The substrate was then soft baked in contact mode for 5 min at 130 °C on a hot plate and cooled to RT. The target film thickness was 60 µm. Due to variability in the processing conditions (temperature, humidity, etc.), our standard operating procedures produced thick films with a ±5 µm thickness around the target specification. Film thickness metrology was performed with a KLA-Tencor (CA, USA) P-15 surface profilometer. We note that in situ EBR is superior to scalpel EBR to best preserve the target spin thickness. This is because as the photoresist edge beads are removed, the resist flows radially into the newly created circumferential voids, reducing the film thickness. For example, when using a scalpel to perform EBR, this mechanism can act more than once, or even several times, significantly reducing the target spin thickness.

The THB-151N application method was used again for the second layer of applied photoresist. The second spin comprised five steps: (1) 300 rpm for 10 s to spread out the photoresist, (2) 0 rpm for 30 s to enable reflow, (3) 800 rpm for 110 s as the thickness-determining step, (4) 300 rpm for 30 s to enable EBR, as was previously described, and (5) 1000 rpm for 2 s to planarize the photoresist. The substrate was then soft baked a second time in contact mode for 5 min at 130 °C on a hot plate and cooled to RT. The final target film thickness of this procedure was 120 µm. We note that simply repeating the first spin step twice would not produce a 120 µm thick film. This result was due to the reduced contact angle at the resist-to-resist interface, as opposed to the resist-to-Cu interface, where van der Waals forces caused the second spin layer to become more viscous than it otherwise would be; thus, the target spin thickness was increased by ~150% (e.g., a 40 µm target second spin thickness is ~60 µm). In addition, an 80 µm target thick film was fabricated with this procedure by using a single spin with a main spin step of 950 rpm for 20 s.

The photoresist was then exposed through a 127 mm^2^ Compugraphics (Glenrothes, Scotland) glass photomask with an 86 mm diameter chrome pattern in a Canon (Tokyo, Japan) PLA600F Mask Aligner using a broad-spectrum USHIO (Livingston, UK) USH-250D super high pressure UV-type mercury lamp with an i-line intensity of 5.3 mW/cm^2^. Aerial exposure doses of 1100 and 1700 mJ/cm^2^ were delivered for the 80 and 120 µm target photoresist film thicknesses, respectively. As a final processing step, the latent image patterned substrate was again attached to the WS-650MZ-23NPPB Laurell photoresist spinner chuck and spin developed. A glass funnel was installed above the substrate, and predefined volumes of JSR Micro TMA238WA TMAOH-based photoresist developer solution were poured onto the rotating photoresist surface, as described in Table 1.

**Table 1:** Spin development procedure used in our experiments

Step	Time (s)	Speed (rpm)	Accel. (rpm/s)	Special	Dispense Type	Quantity (mL)
1	5	100	100	Repeat 2x	Developer	12.5 per pass
2	60	40	100			
3	5	200	100		None	N/A
4	60	200	100	N/A	DI Water	200 total
5	10	300	100			
6	50	500	200		N/A	N/A

Steps 1–3 develop the latent images, and steps 4–6 rinse the film. Steps 1–3 can be repeated as many times as required, depending on the target mold AR and the developer temperature. The listed dispense volume is the minimum to form a continuous puddle on a 100 mm substrate rotating at 40 rpm after an initial wetting volume of 25 mL.

SEM analysis requires a conductive substrate. The complex refractive index, by use of Eqs. (18) and (19)^43^ and assuming *μ*_*r*_ = 1, enabled the determination of the relative permittivity (2.48) and the resistivity of the THB-151N photoresist used in this paper (e.g., 1.79 × 10^8^ Ωcm at a microinductor switching frequency of 10 GHz for granular switch-mode-power-supply (SMPS) integrated voltage regulator (IVR) applications). We note that *n* and *K* were calculated from the THB-151N photoresist Cauchy coefficients.

18 $$n^2 - K^2 = {\it{\epsilon }}_r\mu _r$$

19 $$\sigma = \frac{{2nK{\it{\epsilon }}_0\omega }}{{\mu _r}}$$

To enable SEM imaging, our samples were sputtered with ~10 nm Au/Pt, since THB-151N is an electrical insulator, and scanned with a 10 keV electron beam at a low penetration depth. Aerial and latent image simulations were performed in Wolfram Mathematica v11.1 on a computer with 16 GB Corsair (CA, USA) Vengeance DDR4 2666 MHz RAM, an Intel (CA, USA) Core i5-9400F CPU @ 2.9 GHz (6 cores/threads), an Nvidia (CA, USA) GeForce GTX1660 Super 6 GB GPU and a Samsung (Seoul, South Korea) 970 EVO Plus 500 GB SSD (NVMe M.2 form factor).

## Supplementary information

Supplementary information

## Data Availability

The authors declare that the data supporting the findings of this study are available within the paper.
