# Supplementary material for: Methods for latent image simulations in photolithography with a polychromatic light attenuation equation for fabricating VIAs in 2.5D and 3D advanced packaging architectures
Source: Microsyst Nanoeng. 2021 May 25;7:39. doi: 10.1038/s41378-021-00266-x (PMC8433293; doi:10.1038/s41378-021-00266-x)
Supplement: Supplementary file 1 — Supplementary information [file 41378_2021_266_MOESM1_ESM.docx]

Supplementary information for

**Methods for latent image simulations in photolithography with a polychromatic light attenuation equation for fabricating VIAs in 2.5D and 3D advanced packaging architectures**

Daniel C. Smallwood^1^, Paul McCloskey[^1^](#Author_Affiliations), Cian O’Mathuna[^1^](#Author_Affiliations)^,2^, Declan P. Casey[^1^](#Author_Affiliations) & James F. Rohan[^1^](#Author_Affiliations)

Correspondence: Daniel C. Smallwood (daniel.smallwood@tyndall.ie)

^1^ MicroNano Systems Centre, Tyndall National Institute, University College Cork, Lee Maltings, Cork, Ireland, T12 R5CP

^2^ School of Engineering, University College Cork, College Road, Cork, Ireland

# **List of contents**

**Section S1.** Introducing fast scalar diffraction equations with refraction for apertures and occulters in the near field

**Section S1.1.** Square and rectangular geometries

**Section S1.2.** Circular geometries

**Section S2.** Rapid and accurate latent image calculation with polychromatic exposure including diffraction, reflection, refraction and attenuation

**Section S2.1.** Square and rectangular geometries

**Section S2.2.** Circular geometries

**Section S3.** Validity of near field scalar diffraction equations

**References for this supplementary information**

# **Section S1. Introducing fast scalar diffraction equations with refraction for apertures and occulters in the near field**

### **Section S1.1. Square and rectangular geometries**

We now introduce a succinct set of fast and versatile scalar diffraction equations that include factors for refraction, reflection and angled incidence[^1^](#R1)^,^[^2^](#R2)^,^[^3^](#R3)^,^[^4^](#R4)^,^[^5^](#R5). Equation (1) uses the paraxial approximation to enable calculation of the unattenuated diffracted light intensity at any point (x,y,z) from a rectangular or square aperture. $C\left( \mu_{i},\beta_{i} \right)$ and $S\left( \mu_{i},\beta_{i} \right)$ are the Fresnel integrals (2) and (3) for $\mu_{i}$ and $\beta_{i}$. Equations (4), (5), and (6), (7) are Fresnel numbers for transmission and internal reflection, respectively, including the gap-modified z-factors, $A_{1}$ and $A_{2}$, as described in Fig. 2 of our manuscript, where the refraction angle has been included ($\theta=0$ for vertical incidence) and $\lambda_{1}$ is the wavelength in the first stacked layer.

To include refraction at interface 1 (see manuscript Fig. 2), the radiation path length was modified by the parameter,$g_{2}$ (8), which adjusts $g_{1}$ as the ratio of the refractive index for layer two, $n_{2}$, to the refractive index for layer one, $n_{1}$, where $\delta$ is the incident angle and $\theta$ is the photoresist refraction angle ($cos\delta,\theta=1$ for vertical incidence). Intuitively, this equation states that due to Snell’s law and when reducing the refraction angle, a longer vertical distance is required for the light to traverse the same horizontal distance as the larger incident angle.

The observation plane (the region of interest after the aperture plane) coordinates are mapped by $x$ and $y$, where $x_{i}$ and $y_{i}$ demarcate the horizontal and vertical aperture boundaries, respectively. The $\tan\theta$ term modifies the observation plane coordinates with angled exposure, where plus and minus coefficients are used for positive and negative translations, respectively.

|  | $I_{re}\left( x,y,z \right)=\frac{1}{4}I_{0}(1-R_{i1})(\{\left[ C\left( \mu_{2} \right)-C\left( \mu_{1} \right) \right]^{2}+\left[ S\left( \mu_{2} \right)-S\left( \mu_{1} \right) \right]^{2}\}\times{\{\left[ C\left( \beta_{2} \right)-C\left( \beta_{1} \right) \right]}^{2}+\left[ S\left( \beta_{2} \right)-S\left( \beta_{1} \right) \right]^{2}\}+R_{i2}{\{\left[ C\left( \mu_{4} \right)-C\left( \mu_{3} \right) \right]}^{2}+\left[ S\left( \mu_{4} \right)-S\left( \mu_{3} \right) \right]^{2}\}\times{\{\left[ C\left( \beta_{4} \right)-C\left( \beta_{3} \right) \right]}^{2}+\left[ S\left( \beta_{4} \right)-S\left( \beta_{3} \right) \right]^{2}\})$ | (1) |
| --- | --- | --- |

|  | $C\left( \mu_{i},\beta_{i} \right)=\int_{0}^{\mu_{i},\beta_{i}} cos\left( \frac{\pi}{2}\omega^{2} \right)d\omega, (i=1,2,3,4)$ | (2) |
| --- | --- | --- |

|  | $S\left( \mu_{i},\beta_{i} \right)=\int_{0}^{\mu_{i},\beta_{i}} \sin\left( \frac{\pi}{2}\omega^{2} \right)d\omega, (i=1,2,3,4)$ | (3) |
| --- | --- | --- |

|  | $\mu_{i}=\sqrt{\frac{2\frac{n_{2}}{n_{1}}\cos\theta}{\lambda_{1}\left( z-g_{1}+g_{2} \right)}}[x_{i}-x\pm\left( z-g_{1}+g_{2} \right)\tan\theta], (i=1,2)$ | (4) |
| --- | --- | --- |

|  | $\beta_{i}=\sqrt{\frac{2\frac{n_{2}}{n_{1}}\cos\theta}{\lambda_{1}\left( z-g_{1}+g_{2} \right)}}[y_{i}-y\pm\left( z-g_{1}+g_{2} \right)\tan\theta], (i=1,2)$ | (5) |
| --- | --- | --- |

|  | $\mu_{i}=\sqrt{\frac{2\frac{n_{2}}{n_{1}}\cos\theta}{\lambda_{1}\left( 2T+g_{1}-z+g_{2} \right)}}[x_{i}-x\pm\left( 2T+g_{1}-z+g_{2} \right)\tan\theta], (i=3,4)$ | (6) |
| --- | --- | --- |

|  | $\beta_{i}=\sqrt{\frac{2\frac{n_{2}}{n_{1}}\cos\theta}{\lambda_{1}\left( 2T+g_{1}-z+g_{2} \right)}}[y_{i}-y\pm\left( 2T+g_{1}-z+g_{2} \right)\tan\theta], (i=3,4)$ | (7) |
| --- | --- | --- |

|  | $g_{2}=g_{1}\frac{n_{2}}{n_{1}}\frac{cos\theta}{\cos\delta}$ | (8) |
| --- | --- | --- |

By Babinet’s principle, whereby a bright field mask is replaced with a dark field mask[^6^](#R6)^,^[^7^](#R7)^,^[^8^](#R8), (1) is easily enabled for an occulter, or an opaque diffractor. This is applied by subtracting the amplitude from one prior to squaring the Fresnel integrals in the brackets above.

### **Section S1.2. Circular geometries**

Equations applicable to circular diffractors are next presented. Equations (9) and (11) use the paraxial approximation to enable diffraction amplitude calculations from circular occulters and apertures, respectively[^6^](#R6)^,^[^9^](#R9). As an example, $U_{F_{O}}$ stands for the amplitude, $U$, of an occulter, $O$, in the near field, or Fresnel region, $F$. $V_{n}$ (10) and $W_{n}$ (12) are Lommel functions, $U_{0}$ is the square root of the bulb aerial intensity, $k= 2\pi/\lambda_{1}$ is the wavenumber, $x$ is a radial point on the observation plane, $r$ is the radius, and $u$ and $v$ (13) (14) (15) and (16) are modified Fresnel numbers that include angled incidence and refraction at the photoresist interface, as previously described.

|  | $U_{F_{O}}\left( x,z \right)=U_{0}e^{ikz}e^{\frac{ik}{2z}(x^{2}+r^{2})}[V_{0}(u_{i},v_{i})-iV_{1}(u_{i},v_{i})]$ | (9) |
| --- | --- | --- |

|  | $V_{n}\left( u_{i},v_{i} \right)=\sum_{m=0}^{\infty} \left( -1 \right)^{m}\left( \frac{v_{i}}{u_{i}} \right)^{n+2m}J_{n+2m}(v_{i}), (i=1,2)$ | (10) |
| --- | --- | --- |

|  | $U_{F_{A}}\left( x,z \right)={-U}_{0}e^{ikz}e^{\frac{ik}{2z}(x^{2}+r^{2})}[W_{2}(u_{i},v_{i})-iW_{1}(u_{i},v_{i})]$ | (11) |
| --- | --- | --- |

|  | $W_{n}\left( u_{i},v_{i} \right)=\sum_{m=0}^{\infty} \left( -1 \right)^{m}\left( \frac{v_{i}}{u_{i}} \right)^{-\left( n+2m \right)}J_{n+2m}(v_{i}), (i=1,2)$ | (12) |
| --- | --- | --- |

|  | $u_{1}=\left[ \frac{kr^{2}\frac{n_{2}}{n_{1}}\cos\theta}{\left( z-g_{1}+g_{2} \right)} \right]$ | (13) |
| --- | --- | --- |

|  | $v_{1}=\left[ \frac{kr\frac{n_{2}}{n_{1}}\cos\theta}{\left( z-g_{1}+g_{2} \right)} \right]\left[ x\pm\left( z-g_{1}+g_{2} \right)\tan\theta\right]$ | (14) |
| --- | --- | --- |

|  | $u_{2}=\left[ \frac{kr^{2}\frac{n_{2}}{n_{1}}\cos\theta}{\left( 2T+g_{1}-z+g_{2} \right)} \right]$ | (15) |
| --- | --- | --- |

|  | $v_{2}=\left[ \frac{kr\frac{n_{2}}{n_{1}}\cos\theta}{\left( 2T+g_{1}-z+g_{2} \right)} \right]\left[ x\pm\left( 2T+g_{1}-z+g_{2} \right)\tan\theta\right]$ | (16) |
| --- | --- | --- |

# **Section S2. Rapid and accurate latent image calculation with polychromatic exposure including diffraction, reflection, refraction and attenuation**

### **Section S2.1. Square and rectangular geometries**

Equation (1) can be used to calculate the unattenuated diffraction pattern from a rectangular or square photomask aperture, inclusive of refraction, reflection and angled incidence, but not attenuation. After combining this with the polychromatic light attenuation equation from our manuscript, a succinct diffraction equation (17) is formed, which includes all of the essential latent image simulation parameters: diffraction, reflection, refraction and attenuation.

|  | $I_{{re}_{t}}\left( x,y,z \right)=\frac{1}{4}(I_{A_{1}}\{\left[ C\left( \mu_{2} \right)-C\left( \mu_{1} \right) \right]^{2}+\left[ S\left( \mu_{2} \right)-S\left( \mu_{1} \right) \right]^{2}\}\times{\{\left[ C\left( \beta_{2} \right)-C\left( \beta_{1} \right) \right]}^{2}+\left[ S\left( \beta_{2} \right)-S\left( \beta_{1} \right) \right]^{2}\}+{I_{A_{2}}\{\left[ C\left( \mu_{4} \right)-C\left( \mu_{3} \right) \right]}^{2}+\left[ S\left( \mu_{4} \right)-S\left( \mu_{3} \right) \right]^{2}\}\times{\{\left[ C\left( \beta_{4} \right)-C\left( \beta_{3} \right) \right]}^{2}+\left[ S\left( \beta_{4} \right)-S\left( \beta_{3} \right) \right]^{2}\})$ | (17) |
| --- | --- | --- |

### **Section S2.2. Circular geometries**

By including our polychromatic light attenuation equation, a succinct equation (18) is again formed, which includes all of the essential latent image parameters. This equation is applicable to a circular aperture or occulter.

|  | $I_{F}\left( x,y,z \right)=I_{A_{1}}\left\vert{U_{F}}_{u_{1},v_{1}} \right\vert+I_{A_{2}}\left\vert{U_{F}}_{u_{2},v_{2}} \right\vert$ | (18) |
| --- | --- | --- |

# **Section S3. Validity of near field scalar diffraction equations**

To ensure accurate simulation results using equations (1), (9), (11), (17) and (18), it is critical to understand the paraxial approximation validity range, wherein the aperture/occulter and the observation plane are assumed to be small and well separated. To do this, we derive the Fresnel integral from the full Rayleigh-Sommerfeld integral (approximation-free), which is given by (19)[^10^](#R10),

|  | $U\left( x,y,z \right)=\frac{1}{\lambda}\iint u_{0}(s,t)\left( \frac{1}{kl}-i \right)\frac{z}{l^{2}}e^{ikl}dsdt$ | (19) |
| --- | --- | --- |

where $U$ and $u_{0}$ are the observation and aperture plane amplitudes, respectively, and $l$ is the path length, as given by (20).

|  | $l^{2}={(x-s)}^{2}+{(y-t)}^{2}+z^{2}$ | (20) |
| --- | --- | --- |

First is the Kirchhoff approximation, wherein the aforementioned planes are assumed to be separated by several wavelengths, thus simplifying an integrand amplitude term in (21).

|  | $\frac{1}{kl}-i=\frac{2\pi}{\lambda l}-i\to-i$ | (21) |
| --- | --- | --- |

Equation (22) is found after solving for $l$ and substituting $a=x-s$ and $b=y-t$, which is of the form (23).

|  | $l=z\sqrt{1+\frac{a^{2}+b^{2}}{z^{2}}}$ | (22) |
| --- | --- | --- |

|  | $l=z\sqrt{1+w}$ | (23) |
| --- | --- | --- |

To enable the paraxial approximation, (23) is expanded into the corresponding Taylor series, wherein the first three terms are shown in (24).

|  | $l=z\left( 1+\frac{w}{2}-\frac{w^{2}}{8} \right)$ | (24) |
| --- | --- | --- |

The paraxial approximation is two-part, and firstly entails the zeroth order approximation of (24), which yields $l=z$. This simplifies the remaining amplitude term according to (25).

|  | $\frac{z}{l^{2}}\to\frac{1}{z}$ | (25) |
| --- | --- | --- |

Secondly, the first order approximation yields (26), which transforms the integrand phase term into (27) and results in the Fresnel integral (28). We note that the first order approximation is taken in the phase term due to the wavenumber multiplier, $k$, which is typically$\gg$1.

|  | $l=z\left( 1+\frac{a^{2}+b^{2}}{2z^{2}} \right)$ | (26) |
| --- | --- | --- |

|  | $kl\to kz+\frac{k}{2z}\left( a^{2}+b^{2} \right)$ | (27) |
| --- | --- | --- |

|  | $U\left( x,y,z \right)=\frac{e^{ikz}}{i\lambda z}\iint u_{0}(s,t)e^{\frac{ik}{2z}\left[ \left( x-s \right)^{2}+\left( y-t \right)^{2} \right]}dsdt$ | (28) |
| --- | --- | --- |

The validity standard for (28) is (29)[^11^](#R11), and we are unaware of any literature that explicitly mentions a specific magnitude difference cut-off point.

|  | $z^{3}\gg\frac{\pi}{4\lambda}max\left[ \left( x-s \right)^{2}+\left( y-t \right)^{2} \right]^{2}$ | (29) |
| --- | --- | --- |

To be exact, the paraxial approximation assumes (30), which is required to neglect the second and higher order phase terms. It is critical to verify (30) prior to proceeding with the near field diffraction formulas.

|  | $e^{-ikz\frac{w^{2}}{8}}\approx1$ | (30) |
| --- | --- | --- |

# **References for this supplementary information**

1. Zhu, Z., Zhou, Z.-F., Huang, Q.-A. & Li, W.-H. Modeling, simulation and experimental verification of inclined UV lithography for SU-8 negative thick photoresists. *J. Micromech. Microeng.* **18**, 125017 (2008).

2. Feng, M., Hang, Q-A., Li, W-H., Zhou, Z-F., Zhu, Zhen. Three-dimensional Simulation of the Deep UV Light Intensity Distribution in SU-8 Photoresists. *IEEE*. (2006).

3. Qian, H., Lin, W. & Qi, X. Numerical simulation of Fresnel and Fraunhofer diffractions of monochromatic and white light. *Opt. Eng* **55**, 084104 (2016).

4. Zhou, Z.-F., Shi, L.-L., Zhang, H. & Huang, Q.-A. Large scale three-dimensional simulations for thick SU-8 lithography process based on a full hash fast marching method. *Microelectronic Engineering* **123**, 171–174 (2014).

5. Zhou, Z.-F. & Huang, Q.-A. Modeling and Simulation of SU-8 Thick Photoresist Lithography. in *Microbial Toxins* (eds. Gopalakrishnakone, P., Stiles, B., Alape-Girón, A., Dubreuil, J. D. & Mandal, M.) 1–31 (Springer Netherlands, 2017). doi:10.1007/978-981-10-2798-7_3-1.

6. Harness, A., Shaklan, S., Cash, W. & Dumont, P. Advances in edge diffraction algorithms. *J. Opt. Soc. Am. A* **35**, 275 (2018).

7. Dubra, A. & Ferrari, J. A. Diffracted field by an arbitrary aperture. *American Journal of Physics* **67**, 87–92 (1999).

8. Socha, R. J. *et al.* Contact hole reticle optimization by using interference mapping lithography (IML). in (ed. Smith, B. W.) 222 (2004). doi:10.1117/12.536581.

9. Sommargren, G. E. & Weaver, H. J. Diffraction of light by an opaque sphere 1: Description and properties of the diffraction pattern. *Appl. Opt.* **29**, 4646 (1990).

10. Born, M. & Wolf, E. Principles of Optics, 4th Ed. *Pergamon Press, Oxford.* (1970).

11. Smith, D. G. *Field Guide to Physical Optics*. (SPIE, 2013). doi:10.1117/3.883971.
